# Calculated avoidance: Math anxiety predicts math avoidance in effort-based decision-making

**DOI:** 10.1126/sciadv.aay1062

**Published:** 2019-11-20

**Authors:** Kyoung Whan Choe, Jalisha B. Jenifer, Christopher S. Rozek, Marc G. Berman, Sian L. Beilock

**Affiliations:** 1 Department of Psychology, The University of Chicago, Chicago, IL, USA.; 2 Mansueto Institute for Urban Innovation, The University of Chicago, Chicago, IL, USA.; 3 Department of Psychology, Stanford University, Stanford, CA, USA.; 4 Grossman Institute for Neuroscience, Quantitative Biology and Human Behavior, The University of Chicago, Chicago, IL, USA.; 5 President, Barnard College, Columbia University, New York, NY, USA.

## Abstract

Math anxiety—negative feelings toward math—is hypothesized to be associated with the avoidance of math-related activities such as taking math courses and pursuing STEM careers. However, there is little experimental evidence for the math anxiety-avoidance link. Such evidence is important for formulating how to break this relationship. We hypothesize that math avoidance emerges when one perceives the costs of effortful math engagement to outweigh its benefits and that this perception depends on individual differences in math anxiety. To test this hypothesis, we developed an effort-based decision-making task in which participants chose between solving easy, low-reward problems and hard, high-reward problems in both math and nonmath contexts. Higher levels of math anxiety were associated with a tendency to select easier, low-reward problems over harder, high-reward math (but not word) problems. Addressing this robust math anxiety-avoidance link has the potential to increase interest and success in STEM fields.

## Teaser

Math anxiety predicts how much effort people are willing to put into doing math.

## INTRODUCTION

Individuals who experience mathematics anxiety, or negative feelings and apprehension toward math, are hypothesized to engage in math avoidance behaviors such as taking fewer math-related courses and pursuing fewer science, technology, engineering, and math (STEM)–related occupations than their less math-anxious peers (*1*–*5*). This theoretical link between math anxiety and math avoidance has critical implications for math performance, as it has been suggested to create a vicious cycle that results in limited math practice, poor math performance, increased math anxiety, and additional math avoidance (*6*, *7*). Despite the theoretical importance of math avoidance, however, there is little direct experimental evidence for the math anxiety-avoidance link.

One potential reason for the dearth in experimental research on math anxiety and avoidance is the absence of an empirical, behavioral measure of math avoidance. Previous studies have used math course enrollment (*3*, *8*) and math test–taking strategies (*9*–*11*) as proxies for math avoidance. However, those proxies are confounded by math ability [i.e., it may be that math-anxious individuals avoid difficult math classes because they have low math ability rather than because they have high math anxiety (*3*, *9*)], and their relation with math anxiety has been mixed (*8*–*10*). In short, previous evidence used in support of the math anxiety-avoidance link has been correlational and burdened by interpretation ambiguities common to correlational study designs. Through a novel math avoidance behavioral paradigm, the current study represents the first experimental demonstration of a direct relationship between math anxiety and avoidance, controlling for math ability and other confounds.

Here, we hypothesize that math avoidance is related to individuals’ perceptions of the costs and benefits associated with effortful math engagement and that this perception depends on individual differences in math anxiety. This hypothesis is grounded in research on motivated behavior, which suggests that individuals make decisions based on a series of cost-benefit evaluations regarding task-related effort (*12*). From this perspective, avoidance behavior can be theorized to emerge when an individual perceives the effort-based costs of an action to outweigh the benefits (*13*). Specifically, we hypothesize that math anxiety leads individuals to perceive math to be more effortful and/or less rewarding, thus making them avoid effortful math even when it is incentivized with a high reward. We also hypothesize that the relationship between math anxiety and effort avoidance is exclusive to the context of math since math anxiety should not affect the perception of effort costs and benefits in nonmath contexts. Together, these predictions create our math-specific effort avoidance hypothesis. We preregistered our hypothesis at https://osf.io/9vpgm/ before conducting the confirmatory study.

To test our hypothesis, we measured individuals’ effort avoidance in math and nonmath contexts by using a novel effort-based decision-making task in which cognitive, effort-based costs for solving math and word (spelling) problems were pitted against monetary benefits. Recent studies have used effort-based decision-making tasks to quantify human motivation and examine its relationship to motivational disorders (*13*–*15*), which suggests that this type of paradigm may be particularly advantageous for studying the motivational patterns of individuals who experience math anxiety. Through multiple experiments, we found evidence in support of our math-specific effort avoidance hypothesis: Higher levels of math anxiety were selectively associated with higher levels of effort avoidance in the math condition—but not the word condition—even after adjusting for individual differences in math ability and controlling for related variables such as test anxiety and trait anxiety, which are known to be confounded with math anxiety (*3*, *9*, *10*).

Our paradigm provides a reliable behavioral measure of math avoidance, which fills a major void in the math anxiety literature. Our paradigm can also facilitate the development of effective interventions to break the math anxiety-avoidance link. Addressing math avoidance behaviors can help break the vicious cycle of math anxiety and increase interest and success in STEM fields and is therefore an important topic for future research.

## RESULTS

### Behavioral task and hypothesis

**Fig. 1:**
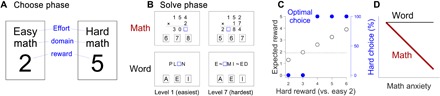
Behavioral task and hypothesis. (**A**) Choose phase of the CAST. In each CAST trial, participants were asked to choose between easy (i.e., low effort) choice cards, which always offered 2 cents, and hard (i.e., high effort) choice cards, which offered one of five possible reward amounts (2, 3, 4, 5, and 6 cents). The domain (either math or word) of the choice cards was kept the same within a trial. (**B**) Four example problems in the solve phase of the CAST. Participants were asked to fill the blue square to make a correct equation (math) or an English word by selecting one of three options below. In word problems, “~” is used in place of some characters to make problems harder. The problems were sorted by seven difficulty levels through a validation study (see the Supplementary Materials). Word answers: PL[A]N, EX[A]MINED. Math answers: 30[8], 2[5]84. (**C**) Expected reward and optimal decision-making as a function of the reward offered in the hard option (the horizontal axis). The horizontal dashed line represents the expected reward (the left vertical axis) of the easy option given the expected accuracy of 95%, and the black open circles represent the expected reward of the hard options, given the expected accuracy of 70%. The blue filled circles indicate the optimal choice probability of the hard options in each reward condition to maximize monetary reward based on expected reward values. (**D**) Math-specific effort avoidance hypothesis. The hard choice probability (HCP; the vertical axis) was defined by averaging the individual HCPs in the 4-, 5-, and 6-cent conditions (C). We predicted that math anxiety would be negatively correlated with the math HCP (red thick line) but not with word HCP (black line).

We collected data across a series of experiments in which participants performed our effort-based decision-making task, the choose-and-solve task (CAST; see tables S1 and S2 for descriptive statistics and correlation matrix of the self-report and behavioral measures). Each CAST trial was either a math or a word trial composed of a “choose,” “solve,” and “feedback” phase. In the choose phase (Fig. 1A), participants were given 3 s to choose between two choice cards, one labeled “easy” and the other labeled “hard.” Easy choice cards always offered a 2-cent reward for a correct response in the subsequent solve phase. Hard choice cards offered one of five possible reward amounts (2, 3, 4, 5, and 6 cents) so that we could obtain a psychometric curve for choosing the hard option as a function of reward and decrease habituation to repeated conditions. After making a selection in the choose phase, participants progressed to the solve phase in which they were given 7 s to solve the corresponding easy or hard math/word problem (Fig. 1B). Participants then progressed to the feedback phase in which they received accuracy feedback on their problem solving (i.e., correct versus incorrect) and were informed of the reward amount they earned for the problem.

**Fig. 2:**
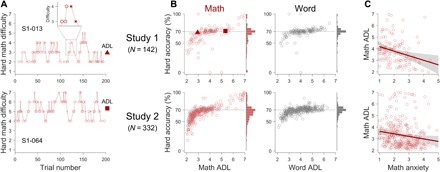
Adaptive difficulty manipulation and validation. (**A**) Time courses of hard math difficulty level from two representative participants. The problem difficulty was determined by the 2-up-1-down staircase procedure (see Materials and Methods). Each circle represents a correct trial, and each × represents an incorrect trial. The difficulty trajectory around trial 120 (indicated with a dashed rectangle) is magnified in the top panel to illustrate the 2-up-1-down staircase procedure. The filled triangle and square symbols on the right indicate the average difficulty level (ADL). (**B**) Relationships between math/word ADL and resulting accuracy in the hard problems. Each circle represents a participant, and the horizontal dotted line at 70% indicates the target accuracy. The filled triangle and square symbols indicate the representative participants in (A). The histograms of the hard accuracy are plotted on the right vertical axes. (**C**) Relationship between math anxiety and math ADL. Each circle represents a participant, the solid lines are the significant regression lines [study 1 (top): Pearson’s *r*(134) = −0.30; 95% CI, −0.45 to −0.14; *P* < .001; study 2 (bottom): *r*(330) = −0.16; 95% CI, −.27 to −.06; *P* = .003], and the gray shades represent the 95% confidence bands.

In the CAST, we reasoned that a rational participant who is trying to maximize earnings would make choices based on the expected values of the easy and hard choice cards (i.e., choose the option with higher expected value), where expected values are determined by the reward at stake and one’s expected accuracy. However, it is possible that less competent participants might also avoid hard options if they experience poor accuracy in solving hard problems. Thus, we aimed to maintain experienced accuracy at a constant level across participants to minimize the possibility that any observed differences in the choice behavior are driven by the variability in problem-solving accuracy arising from differences in participants’ competence. To do so, we sorted a set of 1999 math problems and a set of 1858 word problems based on their solving difficulty (see the Supplementary Materials) and used an adaptive staircase procedure (Fig. 2A; Materials and Methods) to ensure that the difficulty of the hard problems was continuously adjusted in our task to target an accuracy of 70% regardless of participants’ competence. As a result, participant accuracy of the difficulty-adapted hard problems was around 70% [study 1, math: 64.3 ± 17.5% (mean ± SD), word: 68.8 ± 9.3%; study 2, math: 65.3 ± 12.4%, word: 68.5 ± 6.1%; Fig. 2B], and participant accuracy of the easy problems, which were all drawn from the easiest level of our sorting paradigm, was above 90% [study 1, math: 95.4 ± 6.0% (mean ± SD), word: 93.7 ± 6.3%; study 2, math: 93.2 ± 6.9%, word: 92.5 ± 7.2%].

Given these accuracy levels, the hard choice cards that offered more than 3 cents warranted a higher expected value that that of the easy choice cards, which always offered 2 cents (e.g., a hard choice card offering 4 cents with a 70% accuracy rate would hold the expected value of 2.8 cents since 4 × 0.7 = 2.8; open black circles in Fig. 1C). Therefore, to maximize reward in the CAST, one should choose the hard option when it offers more than 3 cents (filled blue circles in Fig. 1C). On the basis of this rationale, we merged trials in which the hard choice card offered 4, 5, and 6 cents and indexed the proportion of these trials in which participants chose the hard option. The proportion of hard choice card selection was also highly correlated across trials that offered 4, 5, and 6 cents (*r* > 0.9), further justifying our decision to merge these three trial types to form one index. This index serves as our dependent variable, which is referred to as participants’ hard choice probability (HCP) and represents the proportion of trials in which participants selected the hard choice card when it was advantageous to do so. We obtained both math and word HCPs from each participant as the CAST included both math and word trials.

We hypothesized that math anxiety would be negatively correlated with the math HCP (red thick line in Fig. 1D) but would not be significantly correlated with word HCP (black line in Fig. 1D), leading to a significant interaction between the math and word conditions. Findings in line with our hypotheses would suggest that math anxiety leads individuals to avoid effortful math even when it is incentivized with a high reward. Such findings would also suggest that the relationship between math anxiety and effort avoidance is exclusive to the context of math. Together, these predictions create our math-specific effort avoidance hypothesis (Fig. 1D).

### Assessing task validity and reliability

Before testing our hypotheses, we assessed the validity and reliability of the CAST-derived variables. Through the adaptive difficulty manipulation (Fig. 2A), we obtained the average difficulty level (ADL) of the hard problems that each participant encountered, which might closely track their performance. Thus, we used math ADL to test the well-known negative association between math anxiety and math performance (*16*–*18*). Consistently, we found that math anxiety was significantly negatively correlated with math ADL [study 1: Pearson’s *r*(134) = −0.30; 95% confidence interval (CI), −0.45 to −0.14; *P* < .001; study 2 (preregistered): *r*(330) = −0.16; 95% CI, −0.27 to −0.06; *P* = 0.003; Fig. 2C], suggesting that ADL is a good proxy for one’s performance. Next, we examined the temporal stability of ADL and HCP within session to assess whether the CAST led to participant fatigue. We reasoned that fatigue would lead to decreased performance and increased effort avoidance, resulting in the decrease in ADL and HCP across blocks and, thus, negative slopes. In contrast, however, the observed slopes of math/word ADL and HCP were significantly positive across blocks (with an exception of study 1 math HCP, which showed a nonsignificant positive slope; fig. S1), suggesting against participant fatigue. Last, we examined the test-retest reliability of ADL and HCP between sessions that were 4 months apart (studies 1 and 1R; see Materials and Methods). We found significant positive test-retest correlations in all math/word ADL and HCP measures [math ADL: Pearson’s *r*(92) = 0.62; 95% CI, 0.47 to 0.73; word ADL: *r*(92) = 0.63; 95% CI, 0.49 to 0.74; math HCP: *r*(101) = 0.64; 95% CI, 0.51 to 0.74; word HCP *r*(101) = 0.68; 95% CI, 0.56 to 0.78; all *P*s < 0.001; fig. S2]. Note that many cognitive tests that are widely used clinically and for research (e.g., working memory tasks) have test-retest correlations that are in the range of 0.3 to 0.7 (*19*, *20*). Together, these results show that the CAST provides reliable measures of performance and choice behavior.

### Testing the math-specific effort avoidance hypothesis

**Fig. 3:**
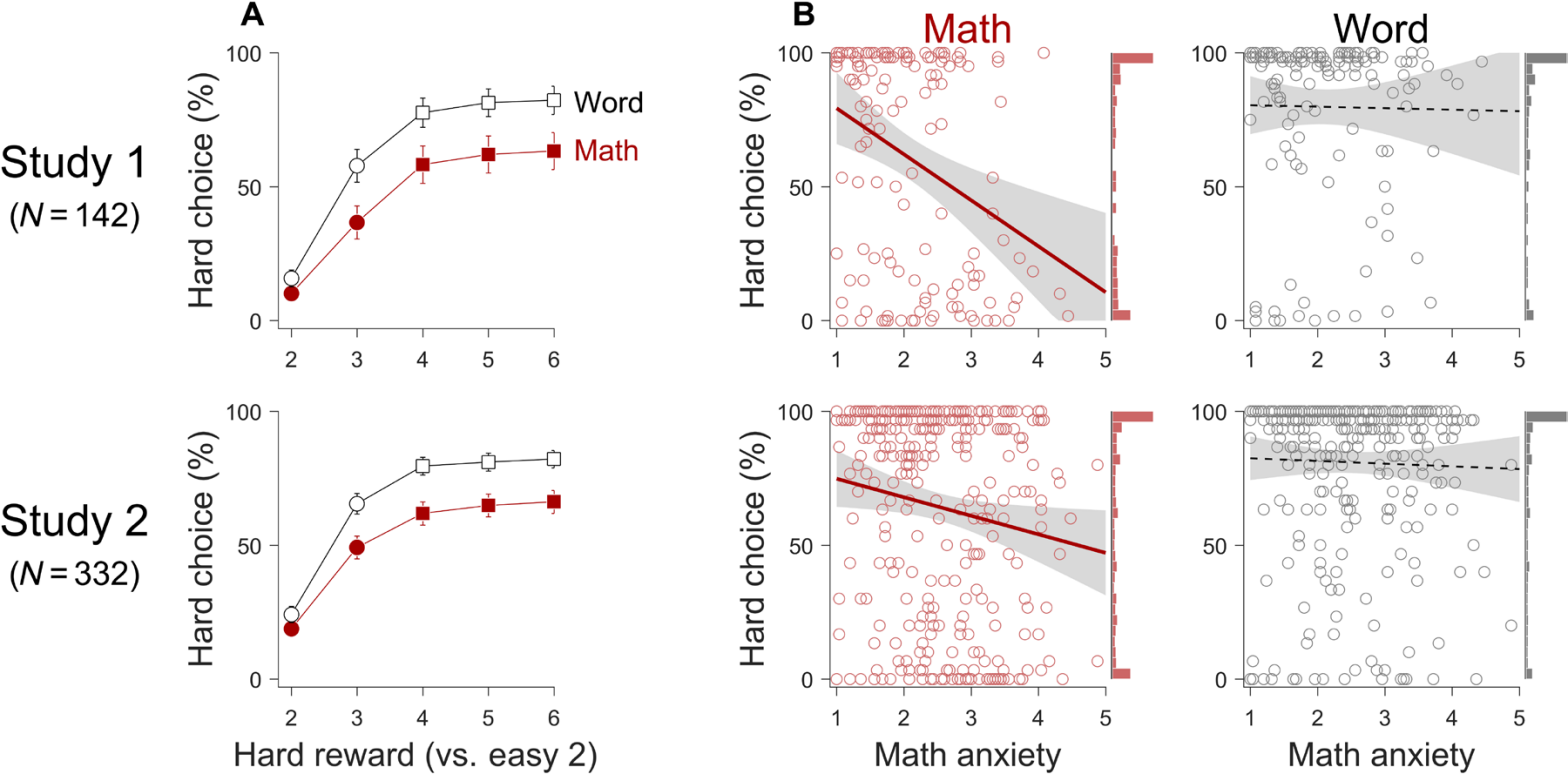
Math-specific effort avoidance. (**A**) Observed HCP as a function of the reward offered in the hard option (the horizontal axis; the easy option always offered 2 cents). The circles and squares specify the HCP in each reward condition averaged across participants, and, especially, the squares represent the conditions in which the hard choice is optimal (see Fig. 1C). Filled red symbols specify the math condition, and open black symbols specify the word condition. Error bars indicate SEM across participants. (**B**) Relationships between math anxiety, math HCP (left), and word HCP (right). Each circle represents a participant, the solid lines are the significant regression lines [the left Math panels; study 1: *r*(140) = −0.34; 95% CI, −0.48 to −0.19; *P* < 0.001; study 2: *r*(330) = −0.15; 95% CI, −0.26 to −0.05; *P* = 0.005], the dashed lines are the nonsignificant regression lines [the right Word panels; study 1: *r*(140) = −0.01; 95% CI, −0.18 to 0.15; *P* = 0.86; study 2: *r*(330) = −0.03; 95% CI, −0.14 to 0.08; *P* = 0.60], and the gray shades represent the 95% confidence bands. The histograms of HCP are plotted on the right vertical axes.

To test the math-specific effort avoidance hypothesis, we conducted an exploratory study (study 1) and examined the relationships between math anxiety and math/word HCP. We found that participants’ HCP generally increased as the reward value for choosing the hard option increased in both the math and nonmath conditions (Fig. 3A). We also found that math anxiety was negatively correlated with math HCP [Pearson’s *r*(140) = −0.34; 95% CI, −0.48 to −0.19; *P* < 0.001; top left in Fig. 3B], but not with word HCP [*r*(140) = −0.01; 95% CI, −0.18 to 0.15; *P* = 0.86; top right in Fig. 3B].

To examine whether the relationship between math anxiety and effort avoidance is exclusive to the context of math, we conducted a linear mixed-effect model (LMM) analysis to test the interaction between domain (math/word) and math anxiety on HCP (Fig. 1D). We controlled for variables related to individual differences in problem solving such as hard versus easy accuracy and response time (RT) since they were correlated with math anxiety (table S1) and could have confounded participants’ choice behavior (see the next section for a more thorough explanation). In this model, the dependent variable was participants’ HCP, and the fixed effects were domain, math anxiety, and their interaction. In addition, participants’ accuracy and RT of easy problems, accuracy and RT of hard problems, and ADL were added as fixed effects for the math and word conditions, respectively. The model (*df* = 267) explained 62.6% of the variance (adjusted *R*^2^); the full results are presented in table S3. We found a significant effect of math anxiety (β = −0.12; 95% CI, −0.19 to −0.05; *P* < 0.001) and a significant interaction between domain and math anxiety on HCP (interaction β = 0.11; 95% CI, 0.04 to 0.18; *P* = 0.001), confirming that the relationship between math anxiety and math HCP significantly differed compared with that between math anxiety and word HCP.

To replicate the findings from study 1, we conducted a confirmatory study with a larger, age-restricted, and gender-balanced sample (study 2) after preregistration (https://osf.io/9vpgm). Again, we found that math anxiety was negatively correlated with math HCP [*r*(330) = −0.15; 95% CI, −0.26 to −0.05; *P* = 0.005; bottom left in Fig. 3B], but not with word HCP [*r*(330) = −0.03; 95% CI, −0.14 to 0.08; *P* = 0.60; bottom right in Fig. 3B]. Since the problem-solving variables in study 2, such as hard versus easy accuracy and RT, were also correlated with math anxiety, even in the word condition (table S2), we conducted an LMM analysis in the same manner as in study 1 to test the math-specific effort avoidance while controlling for the problem-solving variables. The model (*df* = 655) explained 71.6% of the variance (adjusted *R*^2^), and the full results are presented in table S4. Again, we confirmed a significant effect of math anxiety (β = −0.05; 95% CI, −0.09 to −0.00; *P* = 0.03) and a significant interaction between domain and math anxiety on HCP (interaction β = 0.06; 95% CI, 0.02 to 0.10; *P* = 0.003). These results demonstrate that individuals who experience math anxiety choose to avoid exerting greater levels of effort in math, even when it is highly rewarded, and provide a strong evidence for the math-specific effort avoidance hypothesis.

### Examining potential problem-solving confounds on math-specific effort avoidance

Our finding of math-specific effort avoidance may have been confounded by at least two problem-solving behaviors that are less related to math anxiety. First, participants only received rewards when they solved problems correctly, so their problem-solving accuracy may have affected their perceived value of the hard options and their choice behavior. Despite our efforts to hold the accuracy constant with a computer-adaptive paradigm, we observed a significant negative correlation between math anxiety and hard math accuracy (i.e., the higher the math anxiety, the lower the math accuracy; tables S1 and S2, and see fig. S3A for study 2 results). On the basis of this correlation, one could argue that participants with math anxiety avoided hard math because they experienced lower hard math accuracy than their less-math-anxious peers. However, we also observed a significant negative correlation between math anxiety and hard word accuracy in study 2 [*r*(330) = −0.21; 95% CI, −0.31 to −0.11; *P* < 0.001; fig. S3B] but did not observe word effort avoidance (Fig. 3B). Moreover, we confirmed a significant association between math anxiety and math HCP even after controlling for problem-solving variables (tables S3 and S4). These results suggest that the math-specific effort avoidance cannot be explained by differences in problem-solving accuracy.

Second, participants with math anxiety may have avoided hard math problems because they did not expect to solve these problems within the 7 s time limit. We examined math anxiety in relation to the proportion of hard trials in which participants ran out of time while solving problems (fig. S3C) and found that all participants provided responses to most of the hard math problems within the time limit (the proportion of hard math timeout: mean ± SD, 4.4 ± 5.4%; range, 0 to 36.4%) and that the proportion was not significantly correlated with math anxiety in study 2 [*r*(328) = −0.05; 95% CI, −0.16 to 0.06; *P* = 0.39], ruling out this possibility. Moreover, we found that math anxiety was negatively correlated with the amount of time spent on solving hard math problems (i.e., the higher the math anxiety, the shorter the problem-solving time for hard math) [*r*(330) = −0.18; 95% CI, −0.29 to −0.07; *P* = 0.001; fig. S3C]. This suggests that participants with higher levels of math anxiety may have been engaging in even greater math effort avoidance by “guessing” on hard math problems, a speculation that is also supported by the significant negative correlation between math anxiety and hard math accuracy (tables S1 and S2).

### Linking math-specific component of math anxiety and math effort avoidance

Previous research (*3*, *9*, *10*) reported that math anxiety is correlated with other types of anxiety, such as test anxiety and trait anxiety. Similarly, we found that math anxiety was positively correlated with test anxiety, trait anxiety, and reading anxiety in our samples (tables S1 and S2). Thus, it is possible that the observed association between math anxiety and math effort avoidance could be driven by the non–math-specific, general component of anxiety that is shared across math anxiety and other types of anxiety as suggested by previous research (*10*).

Therefore, we tested whether the association between math anxiety and math effort avoidance holds after controlling for other types of anxiety by conducting comprehensive LMM analyses on study 1 (*df* = 116; adjusted *R*^2^ = 41.6%; see table S5 for details) and study 2 (*df* = 321; adjusted *R*^2^ = 47.0%; see Table 1 for details). The variables related to problem solving, such as math ADL and accuracy/RT of easy and hard math, were added as fixed effects, as those were correlated with both math anxiety and math HCP (tables S1 and S2). We confirmed a significant effect of math anxiety on math HCP (study 1: β = −0.19; 95% CI, −0.30 to −0.09; study 2: β = −0.09; 95% CI, −0.14 to −0.04; both *P*s < 0.001) after controlling for these variables, suggesting that math effort avoidance is driven by the math-specific component of math anxiety. We also ran generalized binomial regression analyses to address the nonnormal distribution of math HCP (see the histogram of math HCP in the right vertical axes in Fig. 3B) and confirmed a significant effect of math anxiety on math HCP (study 1: β = −1.22; 95% CI, −2.23 to −0.20; *P* = 0.02; study 2: β = −0.49; 95% CI, −0.92 to −0.03; *P* = 0.03; see tables S6 and S7 for the full results). Together, these findings establish a robust link between math anxiety and math effort avoidance.

**Table 1:** Results of comprehensive LMM analysis for study 2. Dependent variable: math HCP. Independent variables were not transformed. Random effects: The random intercepts for age, gender, education level, highest level of math taken, current math taking, ethnicity, race, and income. All 332 rows (participants) were entered into the model. The maximum likelihood estimation method was used to fit the model. *df* = 321. Adjusted *R*^2^ = 47.04%. The 95% confidence intervals are presented in parentheses beside the βs.

**Predictor**	**β coefficient (95% CI)**	**SE (β)**	***t*(321)**	***P***
Intercept	−0.11 (−0.68 to 0.46)	0.29	−0.38	0.70
Word HCP	0.74 (0.64 to 0.85)	0.05	14.04	<0.001
Math anxiety	−0.09 (−0.14 to −0.04)	0.02	−3.60	<0.001
Reading anxiety	0.05 (−0.00 to 0.09)	0.02	1.93	0.05
Trait anxiety	−0.001 (−0.003 to 0.001)	0.001	−0.81	0.42
Test anxiety	0.003 (0.000 to 0.007)	0.002	2.16	0.03
Easy math accuracy	−0.03 (−0.62 to 0.55)	0.30	−0.12	0.91
Easy math RT	0.01 (−0.06 to 0.08)	0.04	0.22	0.82
Hard math accuracy	0.14 (−0.29 to 0.57)	0.22	0.62	0.53
Hard math RT	−0.03 (−0.08 to 0.02)	0.03	−1.30	0.19
Math ADL	0.08 (0.03 to 0.12)	0.02	3.23	0.001

## DISCUSSION

Math anxiety has long been hypothesized to be associated with math avoidance (*1*–*5*). However, little research has directly investigated this relationship and potential underlying mechanisms, most likely due to the absence of a reliable avoidance measure. We aimed to fill this void in the literature with our novel effort-based decision-making paradigm, the CAST (Figs. 1 and 2). By developing a paradigm in which one can manipulate the levels of effort and reward associated with solving math and nonmath problems, we demonstrated that math anxiety is associated with math-specific effort avoidance over and above math performance (Fig. 3). Moreover, the association between math anxiety and math effort avoidance remained significant after controlling for other types of anxiety (Table 1), suggesting that the math-specific component of math anxiety drives math-specific effort avoidance. Together, these results experimentally establish a robust math anxiety-avoidance link.

Why do individuals with math anxiety avoid exerting effort in math even when it is highly rewarded? Theories of economic decision-making suggest that such avoidance may relate to individuals’ subjective valuation of the effort-based costs and rewards associated with a given task (*12*, *13*). Moreover, it is also possible that individuals with math anxiety reactively avoid math effort because they feel the need to escape, as a spider-phobic would avoid spiders, perhaps due to their highly negative, even traumatic, past experience with math (e.g., failure, humiliation, or the experience of fear). Regardless, we argue that individuals with math anxiety avoid the high-reward, high-effort math options because they perceive the costs of effortful math engagement to outweigh its benefits. Unfortunately, however, this behavioral study cannot differentiate whether math effort avoidance is due to decreased valuation of math-related rewards, greater anticipation of math effort costs, or reactive fear toward math. Moreover, the observed correlation values between math anxiety and math effort avoidance were small to modest (*r*s = −0.37 and −0.15; Fig. 3), suggesting that there are factors other than math anxiety that also affect math avoidance behavior.

How could math anxiety lead to less valuation of reward and/or greater anticipation of cognitive effort in math contexts? Recent research using functional magnetic resonance imaging shows that math anxiety activates the pain network in anticipation of doing math (*21*) and the fear network while either performing math (*22*, *23*) or simply viewing mathematical symbols for a brief period (*24*). These pain- and fear-related neural activations suggest that math anxiety may lead individuals to experience a concrete, visceral sensation of pain and/or fear in math situations, the sensation that may heavily discount the reward associated with math. This speculation is supported by previous research (*25*) demonstrating that pain-associated monetary reward evokes attenuated neural activation in the ventral striatum, the brain region that encodes expected reward (*26*). Simultaneously, regulating such visceral sensation requires substantial cognitive effort (*27*), so it is highly likely that individuals with math anxiety would have to exert cognitive effort in math-related situations (*16*). Consistent with this notion, math-anxious individuals who are able to use greater cognitive control can overcome the adverse effects of math anxiety (*28*).

Among the few differences between studies 1 and 2, it is worth noting that although the observed correlations between math anxiety and math HCP were significant in both studies, the correlation was smaller in study 2 (*r* = −0.15 versus −0.37 in study 1; Fig. 3). Why are the correlation values different? In our analysis of the data from study 1, we found that a few participants completely avoided solving hard problems by always choosing easy options. Realizing that calculating math/word ADL requires participants to solve a minimum number of hard problems, we introduced no-choice trials in study 2 so that participants had to solve at least 10 hard math and 10 hard word problems. It is possible that this forced exposure to hard math problems during the task reduced math effort avoidance in study 2. Consistent with this notion, exposure therapy is known to be effective at reversing avoidance behavior in other forms of anxiety and phobia (*29*). In addition, increasing math exposure through computer games (*30*) or intensive tutoring (*23*) has been shown to provide positive math experiences and improve math performance in students with math anxiety, thereby potentially turning the vicious cycle of math failure➔ increased anxiety➔ increased avoidance➔ increased failure into a virtuous cycle of math success➔ increased confidence➔ increased approach➔ increased success (*7*, *30*).

Choosing to avoid challenging math can start a vicious cycle of math anxiety that results in limited math practice, poor math performance, increased math anxiety, and additional math avoidance (*6*, *7*). Students with math anxiety often choose to take fewer math-related courses and consequently pursue fewer STEM-related occupations than their less-anxious peers (*3*–*5*). By tackling math avoidance early, we may be able to break this vicious cycle before critical academic and occupational choices are made. We envision that our novel task, the CAST, will be used to identify young children and adolescents who have trouble putting effort into math so that targeted interventions can be introduced to reduce their math avoidance and math anxiety. Future research should therefore explore the use of the CAST in more naturalistic settings to validate and optimize its effectiveness for early identification of math effort avoidance.

## MATERIALS AND METHODS

### Experimental design

To assess the relationship between math anxiety and willingness to exert effort in a math and nonmath context, we used questionnaires and a novel effort-based decision-making task, the CAST. Here, we report all experimental conditions, measures, and data exclusion criteria. Studies 1 and 1R were not formally preregistered. The preregistration for study 2 can be accessed at the Center for Open Science (https://osf.io/9vpgm/). The materials, deidentified data, and analysis scripts are openly available at https://osf.io/t4wju/.

### Participants

All studies were approved by the Social and Behavioral Sciences Institutional Review Board of The University of Chicago (IRB no. 16-0639). Participants were recruited via TurkPrime (*31*) to complete the studies online via Amazon Mechanical Turk (see the Supplementary Materials for details) and provided informed consent prior to participation. They were compensated with a combination of base amount and a performance-based bonus in the CAST (described in the “Experimental procedure” section). We limited the analyses to those who passed the problem-solving accuracy criteria (described in the “Choose-and-solve task” section). Outliers were not excluded.

Study 1 was exploratory; a desired sample size of 194 was set to detect an expected correlation of 0.2 with 80% power at a 5% significance level. Because of computer errors, 154 participants completed study 1, and we report the results from 142 participants (age: mean, 37.4; SD, 10.1; range, 21 to 66; sex: 56 females, 77 males, and 9 other/not identified/lost). Study 1R was conducted to measure test-retest reliability of the CAST; 103 of the 142 participants performed the CAST again after 4 months (age: mean, 37.9; SD, 10.1; range, 21 to 66; sex: 41 females, 57 males, and 5 other/not identified/lost). Study 2 was conducted to replicate study 1 on a larger, age-restricted (18 to 35 years old), gender-balanced sample; a sample size of 376 was set to detect a correlation of 0.2 with 95% power at a 5% significance level (i.e., a target sample size of 319) after excluding about 15% of participants who do not pass the preregistered problem-solving accuracy criteria. Of 377 participants who completed study 2, we report the results from 332 participants (age: mean, 28.7; SD, 4.2; range, 19 to 57; sex: 163 females, 165 males, and 4 other/not identified).

### Questionnaires

To measure math anxiety, we administered the short Mathematics Anxiety Rating Scale [sMARS; (*32*)]. Participants responded to questions about how anxious they would feel in different math-related situations (e.g., “signing up for a math course” and “studying for a math test”) on a 5-point Likert scale (1, not at all; 2, a little; 3, a fair amount; 4, much; 5, very much). All analyses were conducted on the average of the 25 items for each participant (Cronbach’s αs = 0.97 for both studies 1 and 2). To isolate math-specific component of math anxiety, we also used an adaptation of the sMARS designed to measure anxiety about reading (e.g., “signing up for an English course” and “studying for an English test”), dubbed the short Reading Anxiety Rating Scale (sRARS; αs = 0.97 for both studies).

To control for other forms of anxiety, we also measured participants’ trait anxiety, test anxiety, and social desirability. Trait anxiety was assessed using the trait component of the State-Trait Anxiety Inventory (*33*), in which participants rated how frequently they experienced feelings of anxiety and calmness (e.g., “I feel nervous and restless” and “I make decisions easily”). Test anxiety was assessed using the Test Anxiety Inventory (*34*), in which participants rated how anxious they feel in 20 test-related situations (e.g., “During tests I feel very tense” and “I feel confident and relaxed while taking tests”). In both measures, items were scored on a 1 to 4 scale and were reverse coded where appropriate. Scores were summed for a composite measure of 20 to 80 (trait anxiety: αs = 0.96 for both studies 1 and 2; test anxiety: αs = 0.96), with a higher value indicating a higher level of trait or test anxiety. Social desirability was measured using the Marlowe-Crowne Social Desirability Scale (*35*) (α = 0.89 for study 1 and α = 0.88 for study 2) to check the underreporting of anxiety (*36*).

We also administered the single-item math/reading anxiety scale (*37*) and the self-math/reading overlap (*38*), the results of which are not reported here. Summary statistics and correlation matrix of self-report and behavioral measures are reported in tables S1 and S2.

### The choose-and-solve task

In the CAST, participants were asked to make a series of binary choices on their willingness to put effort into solving a math or word problem for varying monetary reward. Each CAST trial comprised a choose, solve, and feedback phase.

#### *Choose phase*

Participants first entered the choose phase and were shown a screen containing two choice cards on the left and right sides of the screen (e.g., Fig. 1A); easy choice cards always offered 2 cents, and hard choice cards offered one of five possible reward amounts (2, 3, 4, 5, and 6 cents). The domain (i.e., math or word) of the choice cards was kept the same within a trial. Participants were given 3 s to select a card by pressing one of two designated keys (the “i” key for the choice card seen on the left side of the screen and the “p” key for the choice card seen on the right side of the screen). If they did not make a selection within 3 s, they were automatically directed to an easy problem with 1 cent. The critical dependent measures in the CAST were the hard math and word choice probabilities (math and word HCPs; i.e., probability of choosing the hard choice cards that offer more than 3 cents) because choosing the hard choice cards that offer more than 3 cents was always in the participants’ best financial interest (see Fig. 1C).

#### *Solve and feedback phases*

Participants then entered the solve phase, in which they were given 7 s to solve a three-alternative problem (e.g., Fig. 1B) based on their selection of the choice card in the choose phase (i.e., easy or hard). The problem was drawn from a large pool of problems that were sorted by seven difficulty levels through a prior validation study (see the Supplementary Materials for details). When participants chose the easy card, problems in the easiest level were given. The difficulty of hard problems was continuously calibrated to a target accuracy of 70% regardless of participants’ competence using a 2-up-1-down staircase method; the difficulty level increased after two successive correct trials, with the maximum level of 7 (the hardest level), and decreased after one incorrect trial, with the minimum level of 2 (see Fig. 2A for two exemplar participants). Capitalizing on the adaptive difficulty calibration, the math and word difficulty levels (ADLs) were defined as the average level of the hard problems that one encountered.

In math trials, participants were presented with a multidigit multiplication problem whose solution was missing one digit. They were provided with an answer bank of three digits and were given 7 s to select the missing digit from the three options. In word trials, participants were provided with common English words with one letter removed. Again, they were provided with an answer bank of three letters and were given 7 s to select the missing letter from the three options to complete the word. Participants made their selection by pressing one of three designated keys (“i,” “o,” and “p” for the left, middle, and right options, respectively). To discourage participants from making quick guesses, key responses were not registered by the paradigm until 1.5 s after problem onset. After participants entered their selection or the time was up, the correct answer was displayed along with the number of remaining trials, and if correct, the reward offered was added to the total reward. Participants then proceeded to the next trial at their own pace by pressing the enter or space key.

The problem-solving accuracy criteria were based on participants’ performance in easy math and word problems, which were expected to yield over 90% accuracy; participants whose accuracy was below 70% in either easy math or word problems were excluded from the analysis because their perception of expected rewards was assumed to be very different from the majority of participants, whose accuracy was over 90%. We preregistered these exclusion criteria in study 2.

#### *No-choice trials*

Calculating the math/word competence and easy problem accuracy requires participants to solve a minimum number of both hard and easy problems. However, in study 1, seven participants never chose the hard choice card, and two participants never chose the easy choice card. To address this issue, in study 2, we introduced the no-choice trials in which only one choice card was presented during the choose phase. In these trials, participants were instructed to accept the single choice card by pressing the key corresponding to the side of the screen that the card was presented on (i.e., “i” for left and “p” for right); unlike in the choice trials, the solve phase of these no-choice trials did not begin until they pressed the corresponding key. In study 2, 40 no-choice trials were included (10 easy math, 10 hard math, 10 easy word, and 10 hard word); easy choice cards were always valued at 2 cents, and hard choice cards were always valued at 5 cents. These no-choice trials ensured that participants encountered a minimum number of both easy and hard problems throughout the task that could be used to calculate their math/word competence and easy problem accuracy.

### Experimental procedure

Each study was uploaded as a human intelligence task on Amazon Mechanical Turk to be completed in one session. After providing informed consent, participants first completed a series of questionnaires in the following order: math anxiety (sMARS), reading anxiety (sRARS), reading motivation questions, math motivation questions, self-math/reading overlap, single-item math/reading anxiety, trait anxiety, test anxiety, and social desirability. Ten attention check questions were embedded throughout the questionnaires; if participants missed more than two attention checks, the study session was terminated. Demographics such as gender, age, and race were collected after participants completed questionnaires and the CAST.

Participants then performed the CAST, which started with practice blocks to train them on the problem format, button keys, and time restrictions. The first two practice blocks were designed to familiarize participants with the solve phase of task: All participants solved the same 12 math problems in the first block and the same 12 word problems in the second block (with the presentation order fixed in ascending difficulty). The third practice block mirrored the design of the main CAST blocks (20 trials in studies 1 and 1R; 28 trials in study 2), although no monetary reward was given for performance during practice. In the main portion of the CAST, in which the performance-based bonus was determined, participants in study 1 completed two blocks that each contained 50 math choice trials and 50 word choice trials (200 trials total), participants in study 1R completed five blocks that each contained 10 math choice trials and 10 word choice trials (100 trials total), and participants in study 2 completed five blocks that each contained 10 math choice trials, 10 word choice trials, 4 no-choice math trials, and 4 no-choice word trials (140 trials total). The location of the choice cards (left versus right) was counterbalanced within each block. The difficulty calibration for hard problems began in the practice CAST block and continued through the main blocks. The initial difficulty level was set at level 4 and was adjusted on a trial-by-trial basis according to one’s performance; as a result, each participant may have encountered different problems during the practice CAST block and afterward. The CAST was implemented in jsPsych (*39*), so that it could be administered over the Internet and run on the participants’ web browsers. A working version of the CAST used in study 2 (including the practice blocks) can be found at https://kywch.github.io/CAST_jsPsych/choose-and-solve-task.html.

Each session of study 1 comprised the questionnaires, the practice blocks, 200 trials of the CAST without the no-choice trials, posttask questions, a perceptual metacognition task (*40*) (not reported here), and demographic questions. Participants were paid the base of $1.50 plus performance-based bonus of up to $8.00 (total: mean, $7.55; SD, $0.62). Each study 1R (i.e., the retest of study 1) session comprised the CAST and posttask questions only: 100 trials of the CAST without the no-choice trials and 100 trials of the pilot version of modified CAST (not reported here). Participants were paid the base of $3.00 plus performance-based bonus of up to $8.80 (total: mean, $9.56; SD, $0.74). Each study 2 session comprised the questionnaires, 140 trials (including 40 no-choice trials) of the CAST, posttask questions, and demographic questions. Participants were paid the base of $2.00 plus performance-based bonus of up to $5.40 (total: mean, $5.46; SD, $0.58). The pdf version of the study 2 Qualtrics survey is available at https://osf.io/t4wju/.

### Statistical analysis

All statistical tests were performed using MATLAB R2015b. The corrcoef function was used to calculate the effect size (95% CI) of Pearson’s correlation, the polyfit and polyconf functions were used to calculate the 95% confidence band of a regression, and the anova1 function was used to calculate the *F* statistics. Cronbach’s alpha was calculated using the CronbachAlpha function, which can be obtained from https://www.mathworks.com/matlabcentral/fileexchange/38320. To perform the participant-level LMM analyses, the fitlme function in the Statistics and Machine Learning Toolbox was used, which allows incorporating random effects. The LMM results were replicated with a logistic regression (i.e., the binomial distribution was specified for the math HCP) using the fitglm function. The independent variables were not transformed.

## Supplementary Material

http://advances.sciencemag.org/cgi/content/full/5/11/eaay1062/DC1

Download PDF

Calculated avoidance: Math anxiety predicts math avoidance in effort-based decision-making

## SUPPLEMENTARY MATERIALS

Supplementary material for this article is available at http://advances.sciencemag.org/cgi/content/full/5/11/eaay1062/DC1 Materials and Methods S1. Participants: Recruitment details Materials and Methods S2. The CAST: Creation and validation of the problem set Fig. S1. Temporal stability of the math/word ADLs and HCPs. Fig. S2. Test-retest reliability of the math/word ADLs and HCPs. Fig. S3. Relationships between math anxiety and problem-solving variables. Table S1. Descriptive statistics and correlation matrix of questionnaires and behavioral measures of study 1. Table S2. Descriptive statistics and correlation matrix of questionnaires and behavioral measures of study 2. Table S3. Results of study 1 LMM analysis for the math-specific effort avoidance. Table S4. Results of study 2 LMM analysis for the math-specific effort avoidance. Table S5. Results of comprehensive LMM analysis for study 1. Table S6. Results of confirmatory generalized regression analysis for study 1. Table S7. Results of confirmatory generalized regression analysis for study 2.
