# Supplementary material for: Calculated avoidance: Math anxiety predicts math avoidance in effort-based decision-making
Source: Sci Adv. 2019 Nov 20;5(11):eaay1062. doi: 10.1126/sciadv.aay1062 (PMC6867883; doi:10.1126/sciadv.aay1062)
Supplement: http://advances.sciencemag.org/cgi/content/full/5/11/eaay1062/DC1 [file supp_5_11_eaay1062__index.html]

Science Advances | Science AdvancesAAASSearchScience AdvancesMenu

## Supplementary Materials

**This PDF file includes:**

- Materials and Methods S1. Participants: Recruitment details
- Materials and Methods S2. The CAST: Creation and validation of the problem set
- Fig. S1. Temporal stability of the math/word ADLs and HCPs.
- Fig. S2. Test-retest reliability of the math/word ADLs and HCPs.
- Fig. S3. Relationships between math anxiety and problem-solving variables.
- Table S1. Descriptive statistics and correlation matrix of questionnaires and behavioral measures of study 1.
- Table S2. Descriptive statistics and correlation matrix of questionnaires and behavioral measures of study 2.
- Table S3. Results of study 1 LMM analysis for the math-specific effort avoidance.
- Table S4. Results of study 2 LMM analysis for the math-specific effort avoidance.
- Table S5. Results of comprehensive LMM analysis for study 1.
- Table S6. Results of confirmatory generalized regression analysis for study 1.
- Table S7. Results of confirmatory generalized regression analysis for study 2.

Download PDF

**Files in this Data Supplement:**

- Adobe PDF - aay1062\_SM.pdf
