# Supplementary material for: Calculated avoidance: Math anxiety predicts math avoidance in effort-based decision-making
Source: Sci Adv. 2019 Nov 20;5(11):eaay1062. doi: 10.1126/sciadv.aay1062 (PMC6867883; doi:10.1126/sciadv.aay1062)
Supplement: Download PDF [file aay1062_SM.pdf]

Science Advances
AAAS

advances.sciencemag.org/cgi/content/full/5/11/eaay1062/DC1

# Supplementary Materials for

## Calculated avoidance: Math anxiety predicts math avoidance in effort-based decision-making

Kyoung Whan Choe, Jalisha B. Jenifer, Christopher S. Rozek, Marc G. Berman, Sian L. Beilock*

*Corresponding author. Email: beilock@uchicago.edu

Published 20 November 2019, *Sci. Adv.* **5**, eaay1062 (2019)
DOI: 10.1126/sciadv.aay1062

**This PDF file includes:**

Materials and Methods S1. Participants: Recruitment details
Materials and Methods S2. The CAST: Creation and validation of the problem set
Fig. S1. Temporal stability of the math/word ADLs and HCPs.
Fig. S2. Test-retest reliability of the math/word ADLs and HCPs.
Fig. S3. Relationships between math anxiety and problem-solving variables.
Table S1. Descriptive statistics and correlation matrix of questionnaires and behavioral measures of study 1.
Table S2. Descriptive statistics and correlation matrix of questionnaires and behavioral measures of study 2.
Table S3. Results of study 1 LMM analysis for the math-specific effort avoidance.
Table S4. Results of study 2 LMM analysis for the math-specific effort avoidance.
Table S5. Results of comprehensive LMM analysis for study 1.
Table S6. Results of confirmatory generalized regression analysis for study 1.
Table S7. Results of confirmatory generalized regression analysis for study 2.

**Materials and Methods S1. Participants: Recruitment details**

As indicated in the main text, participants were recruited using TurkPrime to complete the online study via the online labor market Amazon Mechanical Turk (AMT) in which workers perform human intelligence tasks (HITs) for requesters. The study was conducted during weekdays only; participation was allowed only between 8 a.m. to 8 p.m. CST. When participants failed more than two (out of ten) attention check questions embedded in the questionnaires that preceded the choose-and-solve task (CAST), the study session was terminated.

In Study 1, AMT worker qualifications included location in the United States, a HIT approval rate greater than or equal to 98%, and the number of HITs approved greater than or equal to 5,000. A target sample size of 194 was set to detect an expected correlation of .2 between self-reported and behavioral measures with 80% power at a 5% significance level. However, only 154 participants were able to complete the study due to computer errors, and demographic information from 9 (out of 154) participants was lost due to computer errors.

In Study 2, TurkPrime Panel Options were used to recruit specific demographic groups without asking for demographic information before the questionnaires and CAST to avoid triggering stereotypes that could affect the self-report and behavioral measures. Specifically, we targeted 188 female and 188 male participants who speak English as their first language and are between the ages of 18 and 35 in addition to using the AMT worker qualifications of location in the United States, a HIT approval rate greater than or equal to 90%, and the number of HITs approved greater than or equal to 500.

**Materials and Methods S2. The CAST: Creation and validation of the problem set**

*Math problems:* To create a large pool of 3-alternative choice math problems, candidate math problems were first generated by multiplying a list of 3-digit numbers (100-999) with a list of 1-digit numbers (2-9), and by multiplying a different list of 3-digit numbers (100-299) with a list of 2-digit numbers (11-19). A new list of 3x1-digit problems whose solutions did not exceed 3000 and 3x2-digit problems whose solutions did not exceed 6000 were compiled, and 1500 problems from this set were randomly selected for further validation. The set of 1500 problems was adapted to fit our paradigm by removing one digit from the solution of each problem. The

missing digit, which could be the 1st, 2nd, or 3rd digit in the solution, was then used as one of the 3 alternative choices participants could select to solve the problem. An attempt was made to balance the frequency with which digits 0-9 were removed across problems. The other two alternative choices were always one unit higher and one unit lower than the removed digit (i.e. if "2" was the removed digit, the 3-alternative choice options would be "1, 2, 3").

*Word problems:* A list of candidate word-spelling problems were first generated from a list of 350,000 English words downloaded from https://github.com/dwyl/english-words; all words that were 4-13 characters long that had the letters *a, e, i, n, r, or t* as their third letter were selected for testing. Words with common prefixes (i.e., *tra-, cha-, gra-, pre-, ove-, uni-, con-, thr-, per-, for-, str-, par-, mar-, sur-, int-, ret-, aut-, ent-, out-. ext-*, and *ant-*) were removed from the list. Word-spelling problems were then created by replacing the third letter of each word with a blank space. Additional letters of some words were replaced by a tilde (~) to make it harder to determine which letter should go in the blank space. Tildes replaced the second letter of words that were 5-7 characters long (e.g., EVENTS → E~☐NTS), both the second letter and the sixth letter of words that were 8-10 characters long (e.g., EVIDENCE → E~☐DE~CE), and the sixth and ninth letters of words that were 11-13 characters long (e.g., ANNOUNCEMENTS → A~☐ OU~CE~ENTS). Words that became the same after having letters replaced with tildes were removed from the testing set. The solution to the word-spelling problem, which was always the third letter of the word (which we replaced by the blank space), was included as one of the three alternative choices participants could select to solve the problem; the 3-alternative choice sets were always either (*a, e, and i*; if the solution was either a, e, or i) or (*n, r, t*; if the solution was either n, r, or t), presented in this order. All letters in the word-spelling problems were presented in capital letters.

*Problem set validation:* Candidate math and word problems were grouped into sets of 60 to create 25 sets of math problems and 31 sets of word problems. Word problems were grouped so that 10 problems for each letter solution (*a, e, i, n, r, and t*) were included in the set. The positioning (left, middle, right) of the problem's solution in the solution bank was balanced across the problems in each math and word set. Each problem had to be solved within 8 seconds, however, key responses were not registered by the paradigm until 2 seconds after problem onset

to discourage participants from making quick guesses. These sets were then uploaded to AMT as 56 separate HITs (25 math and 31 word HITs) that paid $1.50 each. A maximum of two math HITs and two word HITs were uploaded per day.

50 AMT workers were recruited to complete first ten of the math and word HITs, and 30 workers were recruited to complete the remaining 15 math HITS and 21 word HITs. AMT worker qualifications included location in the United States, a HIT approval rate greater than or equal to 98%, and number of HITs approved greater than or equal to 5,000. Workers provided informed consent before completing the HITs. Data from workers who got less than 28 (out of 60) problems were discarded. 692 unique workers completed at least one HIT (333 unique math workers, 545 unique word workers); 24 workers completed 10 or more math HITs, and 14 workers completed 14 or more word HITs. Workers' response time and accuracy were used to sort 1,999 math problems and 1,858 word problems into seven difficulty levels, which are available at https://osf.io/t4wju/.

| | **Difficulty** | **Level 1** | **Level 2** | **Level 3** | **Level 4** | **Level 5** | **Level 6** | **Level 7** |
|---|---|---|---|---|---|---|---|---|
| Math | **Number** | 499 | 239 | 219 | 224 | 228 | 230 | 360 |
| | **Accuracy (M±SD %)** | 98.1±1.9 | 92.3±3.6 | 85.9±3.1 | 76.5±4.3 | 63.5±5.1 | 49.3±5.0 | 33.5±7.3 |
| | **RT (M±SD s)** | 3.18±0.44 | 3.72±0.36 | 4.07±0.33 | 4.33±0.41 | 4.51±0.45 | 4.72±0.39 | 4.99±0.59 |
| Word | **Number** | 480 | 229 | 230 | 230 | 229 | 230 | 230 |
| | **Accuracy (M±SD %)** | 96.9±2.5 | 89.6±2.6 | 82.8±3.3 | 74.2±3.4 | 64.8±4.2 | 52.8±4.9 | 34.7±9.1 |
| | **RT (s)** | 2.99±0.46 | 3.28±0.43 | 3.62±0.40 | 3.79±0.42 | 4.14±0.42 | 4.47±0.40 | 4.69±0.43 |

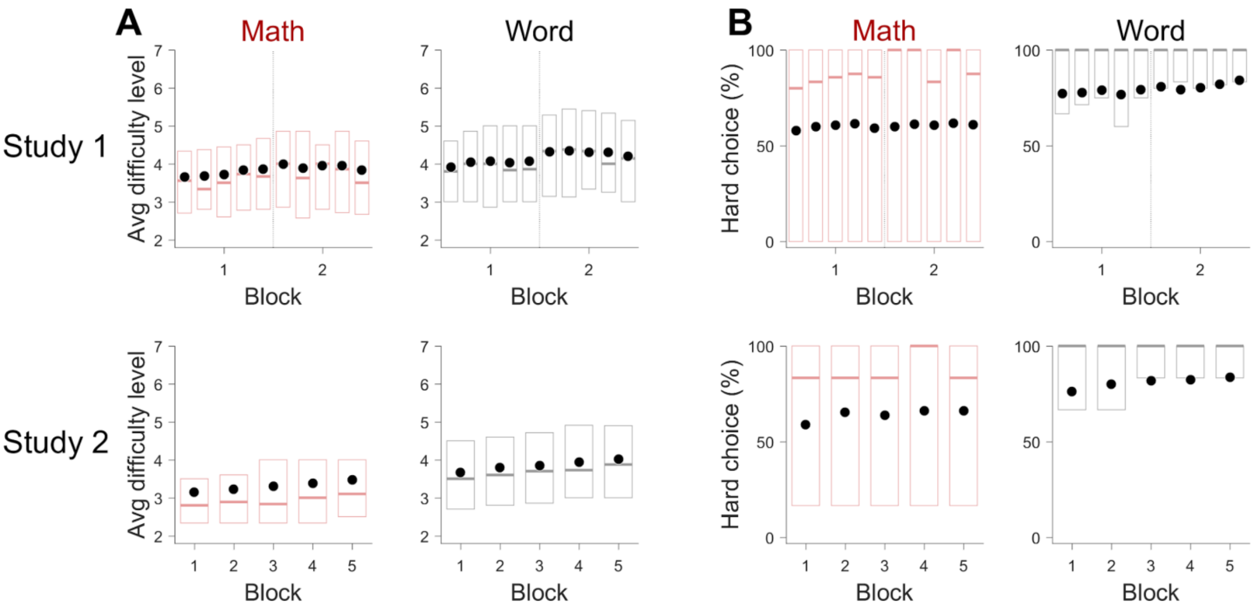


**Fig. S1. Temporal stability of the math/word ADLs and HCPs.** (**A**) Block-by-block time-courses of the math/word averaged difficulty levels (ADLs). In Study 1 (top panels), each block was broken down into five sub-blocks here to make the number of choose-and-solve trials comparable to that in Study 2 (bottom panels). The upper and lower horizontal edges of the rectangles specify the first and third quartile, the middle thick lines specify the median, and the filled circles represent the mean. In all conditions, the slopes were significantly positive (Study 1 math: $\beta$ = .04, 95% CI [.02, .05]; Study 1 word: $\beta$ = .04, 95% CI [.02, .06]; Study 2 math: $\beta$ = .08, 95% CI [.06, .10]; Study 2 word: $\beta$ = .09, 95% CI [.06, .11]; all *p*s<.001). $\beta$ represents ADL change per sub-block (Study 1) or per block (Study 2). (**B**) Block-by-block time-courses of the math/word hard choice probabilities (HCPs). Except the Study 1 math HCP ($\beta$ = 0.2, 95% CI [-0.1, 0.6], *p* = .14), the slopes were significantly positive (Study 1 word: $\beta$ = 0.7, 95% CI [0.4, 1.0]; Study 2 math: $\beta$ = 1.5, 95% CI [0.9, 2.1]; Study 2 word: $\beta$ = 1.7, 95% CI [1.1, 2.3], all *p*s<.001). $\beta$ represents HCP (%) change per sub-block (Study 1) or per block (Study 2).

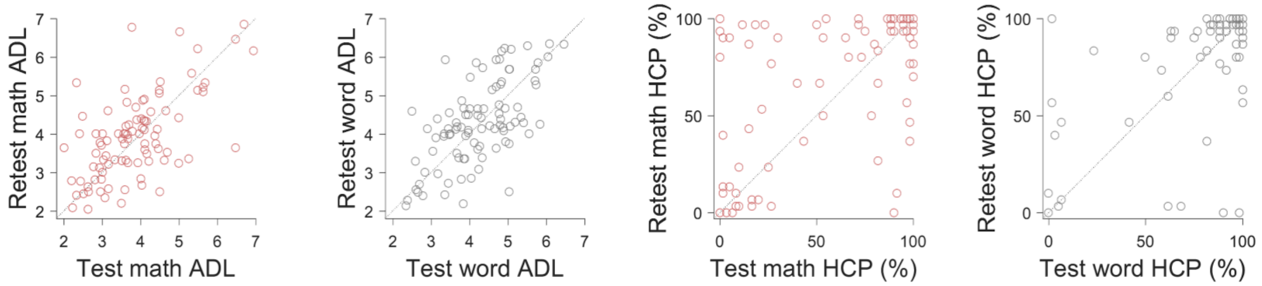


**Fig. S2. Test-retest reliability of the math/word ADLs and HCPs.** The test results (x-axis) are from Study 1, and the retest results (y-axis) are from Study 1R, which was conducted 4 months later by inviting the same participants. Each circle represents a participant, and the dotted line is the identity line.

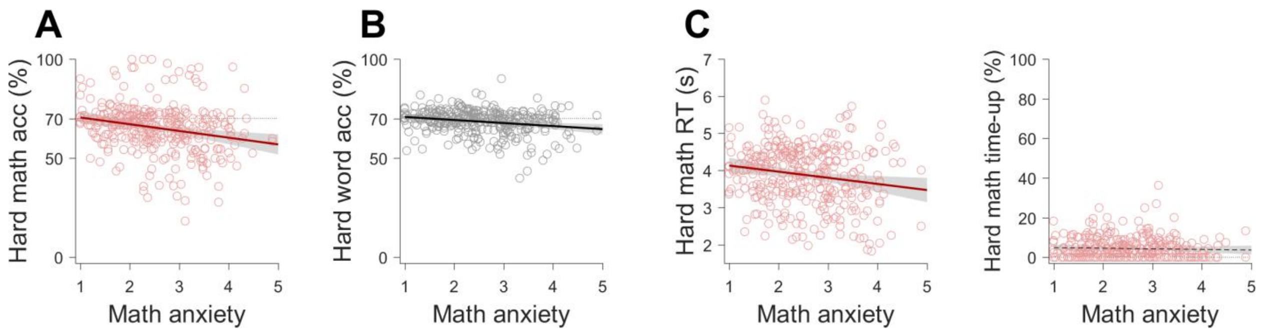


**Fig. S3. Relationships between math anxiety and problem-solving variables.** The results are from Study 2. (**A**) Relationship between math anxiety and hard math accuracy. Each circle represents a participant, the solid line represents a significant correlation (Pearson's $r(330) = -.23$, 95% CI [-.33, -.13], $p < .001$), and the gray shades represent the 95% confidence bands. (**B**) Relationship between math anxiety and hard word accuracy. The correlation was significant, $r(330) = -.21$, 95% CI [-.31, -.11], $p < .001$. (**C**) Relationships between math anxiety and hard math response time (RT) and timeout (in which the response was not entered within the 7-second time limit), respectively. Note that 7 seconds was entered as the RT of the timeout trials. Hard math RT was significantly correlated with math anxiety, $r(330) = -.18$, 95% CI [-.29, -.07], $p = .001$. The proportion of hard math timeout trials was not significantly correlated with math anxiety, $r(328) = -.05$, 95% CI [-.16, .06], $p = .39$. The degrees of freedom are lower in the timeout trial analysis because the trial-by-trial data of two participants were lost.

**Table S1. Descriptive statistics and correlation matrix of questionnaires and behavioral measures of study 1.**

| | Measure | M (SD) | 1 | 2 | 3 | 4 | 5 | 6 | 7 | 8 | 9 | 10 | 11 | 12 | 13 | 14 | 15 | 16 |
|---|---|---|---|---|---|---|---|---|---|---|---|---|---|---|---|---|---|---|
| Self-reports | 1 Math anxiety | 2.09 (0.82) | | | | | | | | | | | | | | | | |
| | 2 Reading anxiety | 1.87 (0.72) | 0.51** | | | | | | | | | | | | | | | |
| | 3 Trait anxiety | 37.18 (13.81) | 0.32** | 0.38** | | | | | | | | | | | | | | |
| | 4 Test anxiety | 34.85 (12.95) | 0.76** | 0.54** | 0.48** | | | | | | | | | | | | | |
| | 5 Social desirability | 13.58 (7.10) | -0.02 | -0.07 | -0.38** | -0.08 | | | | | | | | | | | | |
| CAST | 6 Math HCP (%) | 60.5 (40.8) | -0.34** | 0.00 | -0.04 | -0.22* | -0.10 | | | | | | | | | | | |
| | 7 Math ADL† | 3.75 (1.06) | -0.30** | -0.21* | -0.07 | -0.26* | -0.18* | 0.29** | | | | | | | | | | |
| | 8 Easy math accuracy (%) | 95.4 (6.0) | -0.37** | -0.13 | 0.00 | -0.24* | -0.19* | 0.06 | 0.35** | | | | | | | | | |
| | 9 Easy math RT (s) | 2.23 (0.42) | 0.05 | -0.12 | -0.16 | 0.02 | 0.03 | 0.18* | 0.15 | -0.34** | | | | | | | | |
| | 10 Hard math accuracy† (%) | 64.3 (17.5) | -0.35** | -0.14 | -0.05 | -0.23* | -0.14 | 0.40** | 0.42** | 0.26* | 0.09 | | | | | | | |
| | 11 Hard math RT† (s) | 3.79 (0.83) | -0.16 | -0.07 | -0.05 | -0.17 | -0.29** | 0.12 | 0.51** | 0.35** | 0.26* | 0.21* | | | | | | |
| | 12 Word HCP (%) | 79.8 (31.0) | -0.01 | -0.07 | 0.05 | -0.01 | -0.21* | 0.44** | 0.12 | -0.03 | 0.08 | 0.10 | 0.15 | | | | | |
| | 13 Word ADL† | 4.13 (0.90) | -0.21* | -0.24* | 0.04 | -0.22* | -0.18* | 0.01 | 0.18* | 0.38** | -0.12 | 0.09 | 0.25* | 0.23* | | | | |
| | 14 Easy word accuracy (%) | 93.7 (6.3) | -0.20* | -0.10 | 0.05 | -0.16 | -0.22* | 0.14 | 0.25* | 0.46** | -0.10 | 0.27* | 0.28* | 0.11 | 0.43** | | | |
| | 15 Easy word RT (s) | 2.16 (0.31) | 0.00 | -0.10 | -0.19* | -0.06 | 0.11 | -0.04 | 0.02 | 0.03 | 0.34** | -0.05 | 0.24* | -0.10 | -0.20** | -0.25* | | |
| | 16 Hard word accuracy† (%) | 68.8 (9.3) | -0.03 | -0.07 | 0.10 | 0.00 | -0.06 | 0.05 | 0.09 | 0.24* | -0.10 | -0.03 | 0.07 | 0.36** | 0.44** | 0.16 | -0.11 | |
| | 17 Hard word RT† (s) | 3.07 (0.6) | -0.17 | -0.21* | -0.05 | -0.19* | -0.06 | 0.01 | 0.17* | 0.21* | 0.25* | -0.06 | 0.46** | 0.00 | 0.20** | 0.14 | 0.55** | 0.10 |

*Notes.* Correlations values were based on 142 participants who satisfied the problem-solving accuracy criteria (see Materials and Methods). †Correlation values were based on 135 participants, after excluding participants who never chose the hard options in either math or word conditions. *$p < .05$, **$p < .001$.

**Table S2. Descriptive statistics and correlation matrix of questionnaires and behavioral measures of study 2.**

| | Measure | M (SD) | 1 | 2 | 3 | 4 | 5 | 6 | 7 | 8 | 9 | 10 | 11 | 12 | 13 | 14 | 15 | 16 |
|---|---|---|---|---|---|---|---|---|---|---|---|---|---|---|---|---|---|---|
| Self-reports | 1 Math anxiety | 2.52 (0.84) | | | | | | | | | | | | | | | | |
| | 2 Reading anxiety | 1.98 (0.78) | 0.41** | | | | | | | | | | | | | | | |
| | 3 Trait anxiety | 43.1 (14.7) | 0.27** | 0.27** | | | | | | | | | | | | | | |
| | 4 Test anxiety | 41.5 (14.5) | 0.62** | 0.52** | 0.50** | | | | | | | | | | | | | |
| | 5 Social desirability | 15.7 (6.7) | -0.08 | -0.11* | -0.42** | -0.21** | | | | | | | | | | | | |
| CAST | 6 Math HCP (%) | 64.3 (38.2) | -0.15* | 0.07 | 0.05 | 0.05 | -0.07 | | | | | | | | | | | |
| | 7 Math competence | 3.31 (1.13) | -0.16* | 0.07 | 0.05 | 0.01 | -0.14* | 0.33** | | | | | | | | | | |
| | 8 Easy math accuracy (%) | 93.2 (6.9) | -0.23** | 0.05 | -0.06 | -0.08 | -0.06 | 0.11 | 0.27** | | | | | | | | | |
| | 9 Easy math RT (s) | 2.37 (0.60) | 0.09 | 0.00 | 0.05 | 0.13* | 0.01 | 0.14* | 0.37** | -0.36** | | | | | | | | |
| | 10 Hard math accuracy (%) | 65.3 (12.4) | -0.23** | 0.08 | 0.07 | -0.01 | -0.16* | 0.32** | 0.80** | 0.41** | 0.22** | | | | | | | |
| | 11 Hard math RT (s) | 3.88 (0.79) | -0.18* | 0.05 | 0.00 | -0.06 | -0.11* | 0.15* | 0.52** | 0.25** | 0.44** | 0.49** | | | | | | |
| | 12 Word HCP (%) | 80.9 (29.3) | -0.03 | -0.06 | 0.07 | 0.03 | -0.10 | 0.59** | 0.09 | -0.01 | 0.12* | 0.13* | 0.08 | | | | | |
| | 13 Word competence | 3.87 (0.95) | -0.21** | -0.15* | 0.10 | -0.17* | -0.25** | 0.02 | 0.12* | 0.26** | -0.23** | 0.16* | 0.04 | 0.20** | | | | |
| | 14 Easy word accuracy (%) | 92.5 (7.2) | -0.22** | -0.16* | 0.05 | -0.16* | -0.12* | 0.03 | 0.17* | 0.27** | -0.15* | 0.17* | 0.15* | 0.11* | 0.50** | | | |
| | 15 Easy word RT (s) | 2.20 (0.35) | 0.11* | 0.09 | -0.11* | 0.07 | 0.13* | -0.03 | 0.01 | -0.12* | 0.39** | 0.02 | 0.30** | -0.05 | -0.38** | -0.46** | | |
| | 16 Hard word accuracy (%) | 68.5 (6.1) | -0.21** | -0.17* | -0.04 | -0.24** | -0.11* | 0.08 | 0.14* | 0.22** | -0.16* | 0.15* | 0.09 | 0.15* | 0.61** | 0.44** | -0.22** | |
| | 17 Hard word RT (s) | 3.11 (0.56) | -0.11* | -0.04 | 0.00 | -0.06 | -0.03 | 0.02 | 0.16* | 0.16* | 0.21** | 0.20** | 0.53** | 0.08 | 0.22** | 0.11* | 0.46** | 0.10 |

*Notes.* Correlations values were based on 332 participants who satisfied the problem-solving accuracy criteria (see Materials and Methods). In contrast to Study 1, all Study 2 participants encountered at least 10 easy and 10 hard problems in both the math and word conditions throughout the task. *$p < .05$, **$p < .001$.

**Table S3. Results of study 1 LMM analysis for the math-specific effort avoidance.**

| Predictor | ***β*** **coefficient** | **SE(*β*)** | ***t*(267)** | ***p*** |
|---|---|---|---|---|
| Intercept | 0.40 [-0.42, 1.22] | 0.42 | 0.96 | .34 |
| Domain: word | -0.14 [-0.30, 0.02] | 0.08 | -1.77 | .08 |
| Math anxiety | -0.12 [-0.19, -0.05] | 0.04 | -3.41 | < .001 |
| ADL | 0.06 [0.02, 0.10] | 0.02 | 3.08 | .02 |
| Easy accuracy | -0.15 [-0.86, 0.57] | 0.36 | -0.41 | .68 |
| Easy RT | 0.09 [-0.02, 0.21] | 0.06 | 1.66 | .10 |
| Hard accuracy | 0.55 [0.28, 0.83] | 0.14 | 3.97 | < .001 |
| Hard RT | -0.05 [-0.11, 0.01] | 0.03 | -1.59 | .11 |
| Word * math anxiety | 0.11 [0.04, 0.18] | 0.04 | 3.24 | .001 |

| **Random effects covariance parameters** | | | |
|---|---|---|---|
| Group: Participant (141 Levels) | | | |
| Name1 | Name 2 | Type | Estimate |
| Intercept | Intercept | std | 0.22 |

*Notes.* Dependent variable: HCP from the math and word conditions. Random effects: the by-participant random intercepts. Out of 284 rows (142 participants x 2, math and word), 8 rows were dropped because the participant did not solve any hard math or word problems. Thus 276 rows were entered into the model. The maximum likelihood estimation method was used to fit the model. $df = 267$. Adjusted $R^2 = 62.6\%$. 95% confidence intervals are stated beside the *β*s.

**Table S4. Results of study 2 LMM analysis for the math-specific effort avoidance.**

| Predictor | ***β*** **coefficient** | **SE(*β*)** | ***t*****(655)** | ***p*** |
|---|---|---|---|---|
| Intercept | 0.23 [-0.18, 0.64] | 0.21 | 1.08 | .28 |
| Domain: word | -0.03 [-0.14, 0.08] | 0.06 | -0.51 | .61 |
| Math anxiety | -0.05 [-0.09, -0.00] | 0.02 | -2.11 | .04 |
| ADL | 0.07 [0.04, 0.10] | 0.01 | 4.86 | < .001 |
| Easy accuracy | 0.19 [-0.19, 0.57] | 0.19 | 0.98 | .32 |
| Easy RT | 0.01 [-0.05, 0.07] | 0.03 | 0.41 | .68 |
| Hard accuracy | 0.20 [-0.10, 0.50] | 0.15 | 1.30 | .20 |
| Hard RT | -0.01 [-0.05, 0.03] | 0.02 | -0.41 | .68 |
| Word * math anxiety | 0.06 [0.02, 0.10] | 0.02 | 3.03 | .003 |

| **Random effects covariance parameters** | | | |
|---|---|---|---|
| Group: Participant (332 Levels) | | | |
| Name1 | Name 2 | Type | Estimate |
| Intercept | Intercept | std | 0.25 |

*Notes.* Dependent variable: HCP from the math and word conditions. Random effects: the by-participant random intercepts. All 664 rows (322 participants x 2, math and word) were entered into the model. The maximum likelihood estimation method was used to fit the model. *df* = 655. Adjusted $R^2$ = 71.6%. 95% confidence intervals are stated beside the *β*s.

**Table S5. Results of comprehensive LMM analysis for study 1.**

| **Predictor** | ***β* coefficient [95% CI]** | **SE(*β*)** | ***t*(116)** | ***p*** |
|---|---|---|---|---|
| Intercept | 0.17 [-1.13, 1.47] | 0.66 | 0.26 | .79 |
| Word HCP | 0.52 [0.34, 0.69] | 0.09 | 5.92 | < .001 |
| Math anxiety | -0.19 [-0.30, -0.09] | 0.05 | -3.61 | < .001 |
| Reading anxiety | 0.15 [0.06, 0.23] | 0.04 | 3.26 | .001 |
| Trait anxiety | 0.001 [-0.003, 0.005] | 0.002 | 0.53 | .59 |
| Test anxiety | -0.001 [-0.008, 0.006] | 0.004 | -0.36 | .72 |
| Easy math accuracy | -0.49 [-1.71, 0.73] | 0.61 | -0.80 | .43 |
| Easy math RT | 0.18 [0.03, 0.32] | 0.07 | 2.35 | .02 |
| Hard math accuracy | 0.50 [0.15, 0.84] | 0.17 | 2.86 | .005 |
| Hard math RT | -0.06 [-0.15, 0.02] | 0.04 | -1.57 | .12 |
| Math ADL | 0.05 [-0.01, 0.11] | 0.03 | 1.50 | .14 |

*Notes.* Dependent variable: math HCP. Independent variables were not transformed. Random effects: the random intercepts for age, gender, education level, highest level of math taken, current math-taking, ethnicity, race, and income. Out of 142 rows (participants), 15 rows were dropped because the participant did not solve any hard math problems or their demographic information was missing. Thus 127 rows were entered into the model. The maximum likelihood estimation method was used to fit the model. *df* = 116. Adjusted $R^2$ = 41.6%. 95% confidence intervals are stated beside the *β*s.

**Table S6. Results of confirmatory generalized regression analysis for study 1.**

| Predictor | *β* coefficient | SE(*β*) | *t*(124) | *p* |
|---|---|---|---|---|
| Intercept | -5.40 [-17.2, 6.40] | 5.96 | -0.91 | .36 |
| Word HCP | 2.89 [1.30, 4.47] | 0.80 | 3.60 | < .001 |
| Math anxiety | -1.22 [-2.23, -0.20] | 0.51 | -2.37 | .02 |
| Reading anxiety | 1.02 [0.11, 1.94] | 0.46 | 2.22 | .03 |
| Trait anxiety | 0.01 [-0.03, 0.05] | 0.02 | 0.53 | .59 |
| Test anxiety | -0.01 [-0.06, 0.05] | 0.03 | -0.24 | .81 |
| Easy math accuracy | -0.64 [-10.9, 9.60] | 5.17 | -0.12 | .90 |
| Easy math RT | 1.66 [-0.17, 3.49] | 0.92 | 1.79 | .07 |
| Hard math accuracy | 2.93 [-0.45, 6.31] | 1.71 | 1.71 | .09 |
| Hard math RT | -0.58 [-1.34, 0.17] | 0.38 | -1.54 | .12 |
| Math ADL | 0.40 [-0.21, 1.01] | 0.31 | 1.29 | .20 |

*Notes.* Dependent variable: math HCP. Independent variables were not transformed. Out of 142 rows (participants), 7 rows were dropped because the participants did not solve any easy/hard problems. Thus 135 rows were entered into the model. The binomial distribution was specified for the dependent variable. $df = 124$. Adjusted $R^2 = 45.5\%$. 95% confidence intervals are stated beside the *β*s.

**Table S7. Results of confirmatory generalized regression analysis for study 2.**

| Predictor | *β* coefficient | SE(*β*) | *t*(321) | *p* |
|---|---|---|---|---|
| Intercept | -5.01 [-10.6, 0.58] | 2.84 | -1.76 | .08 |
| Word HCP | 4.12 [2.40, 5.29] | 0.60 | 6.90 | < .001 |
| Math anxiety | -0.49 [-0.93, -0.05] | 0.22 | -2.19 | .03 |
| Reading anxiety | 0.38 [-0.06, 0.83] | 0.23 | 1.69 | .09 |
| Trait anxiety | -0.01 [-0.03, 0.02] | 0.01 | -0.59 | .56 |
| Test anxiety | 0.01 [-0.02, 0.04] | 0.01 | 0.94 | .35 |
| Easy math accuracy | 0.57 [-4.69, 5.82] | 2.67 | 0.21 | .83 |
| Easy math RT | 0.35 [-0.55, 1.24] | 0.45 | 0.76 | .45 |
| Hard math accuracy | 0.26 [-3.67, 4.19] | 2.00 | 0.13 | .90 |
| Hard math RT | -0.28 [-0.77, 0.20] | 0.25 | -1.16 | .25 |
| Math ADL | 0.66 [0.17, 1.15] | 0.25 | 2.63 | .008 |

*Notes.* Dependent variable: math HCP. Independent variables were not transformed. All 332 rows (participants) were entered into the model. The binomial distribution was specified for the dependent variable. $df = 321$. Adjusted $R^2 = 46.3\%$. 95% confidence intervals are stated beside the *β*s.
